# The nexus between remittance, exchange rate and economic growth of E7 economies: Frequency domain analysis

**DOI:** 10.1016/j.heliyon.2023.e21554

**Published:** 2023-11-02

**Authors:** Khalid Usman

**Affiliations:** a School of Economics and Management, Guangzhou City Construction College, Guangzhou City, Guangdong Province, China; b School of Business Administration, University of Science and Technology of China, Hefei City, Anhui Province, China

**Keywords:** Remittance, Agriculture, Economic growth, Exchange rate, Trade, E7 economies

## Abstract

This study investigates the nexus between economic growth (GDP), trade openness, remittances, exchange rate, and agricultural output in E7 (Russia, Indonesia, Mexico, China, India, Brazil, and Turkey) economies, covering the data from 1990 to 2020. In the first step, we adopted the Dumitrescu and Hurlin time domain Granger causality test, which shows that economic growth (GDP) and agriculture do not have any causal relationship, not even at 3-lags. However, uni-directional causality exists between agriculture and economic growth at the first level difference. In the second step, we used Granger causality analysis in the frequency domain, presented by Croux and Reusens, showing a bivariate correlation among economic growth, trade, remittance, and agricultural output. The panel Granger causality outcome indicated that the causality between variables with various frequencies differs. Policymakers in these economies may consider these pragmatic results to formulate valuable and appropriate short-, medium-, and long-term economic strategies.

## 1 Introduction

Since the formation of the Bretton Woods system, the exchange rate has become extremely unstable due to the fluctuating exchange rate. Numerous researchers emphasized evaluating how exchange rate strategies affect economic growth and net exports. So, from an increasingly globalized economic environment perspective, policymakers need to interpret these relationships better. The preference for an exchange rate system is also closely aligned with microeconomics, which has attracted extensive attention and impacted trade flows over the last decade [[1], [2], [3], [4], [5], [6], [7], [8]]. Economists have spent much time analyzing the influence of exchange rate volatility on the economy [[9], [10], [11], [12]]. A significant characteristic of the Post-Bretton Wood era was that many economies witnessed a fluctuating exchange rate system, resulting in unpredictable bilateral exchange rate volatility [[12], [13], [14], [15], [16], [17]]. The influence of exchange rate ﬂuctuations supports countries with short levels of fiscal growth [18].

Exchange rate fluctuations have opposing influences on the flow of trade. So, to keep the exchange rate stable, intervention in the global market exchange is considered beneficial for trade and economic growth promotion. However, in economies where exchange rate fluctuations exert an optimistic influence on their trade flow, a higher receptive exchange rate and fewer market interventions will be fruitful relative. The benefit of remittance inﬂux is that it is a topsy-turvy” basis of expansion funds because it is immediately obtained by families, dissimilar to official development assistance (ODA) and Foreign direct investment (FDI), which are acquired through institutions [[19], [20], [21]]. As [22] pointed out, large remittances flowing into emerging economies are more likely to raise the real exchange rate in a country, influencing competitiveness and economic growth subsequently.

### 1.1 Background of E7 economies

Emerging Seven economies (E7), including China, Turkey, Mexico, India, Indonesia, Brazil, and Russia, are no exception to this path because of high remittance inflow. The E7 group of economies, especially China, India, and Mexico, has been the top recipient of remittances in the world since 2018; for example, India has (US dollar 78.6 billion) remittances, following China (US dollar 67.4 billion) and Mexico (US dollar 35.7 billion) [23]. According to the estimates of the World Bank in 2019, the percentage of remittances in the GDP of China, Mexico, Turkey, India, Brazil, Russia, and Italy were 0.1 %, 3.1 %, 0.1 %, 2.9 %, 0.2 %, 0.6 %, and 0.5 % respectively. Although remittances to E7 economies have increased significantly, their influence on private financing and economic growth is still uncertain. Numerous researchers believe that remittances may cause the devouring and operation of other non-trade areas to catch up, thus damaging private financing; this phenomenon is often called Dutch disease [[24], [25], [26], [27], [28]]. Therefore, remittance flow could drive national financing [29].

In 2006, Price water house coopers founded Emerging Seven (E7) Economies, created by Ref. [30] are seven key emerging economies (China, Russia, India, Mexico, Brazil, Turkey, and Indonesia). However, the total gross domestic product of E7 economies was about 55 %, compared with that of the G7 (UK, Canada, Italy, USA， France, Germany, Japan). So, its (E7) GDP is estimated to almost double that of the G7 Statista Research Department (2016) by 2050. In 2014, E7 had bottled up G7 [31] in the context of purchasing power parity (PPP), and it estimates by the end of 2050, the PPP of E7 may boost up to 75 % larger than G7 [32]. It's estimated that by 2030, the nominal GDP (at PPP exchange rates) of six E7 economies (excluding Mexico) will be among the top ten economies [33]. Thereupon, the financial markets of E7 countries are viewed as an important research domain for researchers.

Neocoronal pneumonia, called coronavirus (COVID-19), is one of the most lethal critical lung syndromes. No cure has yet been found for the disease, which transmits through breathing. The infection first appeared in Wuhan, China (Nov. 2020) and spread worldwide due to the lack of measures taken for a long time. The outbreak was attempted to be controlled by blockade [34], resulting in an outflow of foreign tourists and investors with the fear of infections [35]. This affirms the flow of foreign remittances/currencies from the state. Because of the outflow of foreign currencies, the rate of exchange increased. This condition negatively impacted countries facing foreign currency deficits [36].

[37] pointed out that exchange rates function to control business and capital flows by numerous catching-up countries, which often have constant deficits in their account balance. “This is a prerequisite for these emerging economies to achieve stable and viable economic growth. This means that these emerging nations can use the exchange rate depreciation as a normal policy recommendation to ensure macroeconomic stability to a certain extent and maintain the trade deficit by improving their export competitiveness. Therefore, the devaluation of these economies' currencies makes exports cheaper and imports rather costly. Hence, the native currency will have positive effects.

Exchange rate fluctuations frequently measure the reactions of these determinants (variables) to economic growth. For example, the growth of the local currency may lead to a substantial reduction in received remittance. This indicates that exchange rate volatility alerts remittance reception, thus influencing the economy's growth [[38], [39], [40]]. For example, the impact on trade and agriculture [21] can be classified from the exaggerated monetary perspective, which may adversely affect economic growth by reducing export challenges and exerting a significant burden on the current account.

The research continues to debate how the constant flow of overseas remittances to the E7 nations influences currency rates and economic development. The inconsistency in the literature is possibly attributed to the differences in method and challenges in evaluating credible and constant statistics. Although remittances are an essential source of foreign capital inflow and are received regularly, there hasn't been much or no empirical research on how remittances, exchange rates, and economic growth interact in the E7 group of nations. To the best of our knowledge, however, the study based on the E7 nations differs from all the studies mentioned above based on distinct techniques and chosen countries/regions.

Because of this, it's vital to recognize the influence of exchange volatility on growth, focusing on remittance, agriculture, and trade. Therefore, this research used panel causality and frequency domain analysis to explain the heterogeneous panels in the time domain valuation methods to study annual data of E7 economies from 1990 to 2020 to check the variation of the relationship between economic growth (GDP), exchange rate volatility, trade openness, remittance, and agriculture outputs. This research facilitates to work in three aspects.

(i) From methodological context, the influence of the frequency domain will be measured upon the remittance, exchange rate volatility, economic growth (GDP), trade, and agriculture outputs debate;
(ii) Concerning the area of application, we covered more current time than the bulk of the present research;
(iii) It has been observed 1st time that economic growth (GDP) & exchange rate do have a relationship with agriculture outputs, trade, and remittance, which are the leading operators of E7 countries.

To predict our findings, there is extensive evidence regarding bidirectional relations among economic growth, trade, remittance, and agricultural output of E7 economies.

The remainder of this research article is structured as follows: Part 2 delivers the related literature; Part 3 offers the data collection and methodological approaches. Part 4 states the findings and discussion, while Part 5 confers the research conclusion and specific policy implications.

## 2 Literature review

### 2.1 Marshall-Lerner theory

The traditional perception of depreciation of the national currency to expand the balance of trade is based on the partial equilibrium approach and static of the international balance of payments called the elasticity approach. Primarily, local money depreciation induces local goods into the global marketplace, leading to increased exports, while global goods are expensive in the local bazaar, leading to reduced imports. Both of these influences have led to the improvement of the native trade balance. This approach stems from 'Lerner's work; the influence of the rate of exchange on economic growth is top assessed via the interaction of export subsidies, import quotas, and tariffs [41,42]. The Marshall-Lerner (ML) condition stipulates that devaluation shows a positive shock on the trade balance; the ratio of the exchange rate elasticity of exports and imports must exceed 1 % of the absolute value [43]. According to ML terms, the foreign exchange market remains indirectly constant. This is due to the reason that when the rate of exchange is higher than the equilibrium level, surplus foreign exchange will occur, and vice versa. The ML condition is an empirical study of long-term (equilibrium) conditions by understanding the reaction of the export and import level variables to exchange rate changes [44]. The proposition of [45] was amended by Marshall-Lerner's condition that highlighted the practice of rate of exchange as a strategy tool to operate the trade balance, according to the [46] price elasticity of demand theory [[47], [48], [49], [50], [51]]. This situation pointed out that the domestic economy adopts strategic devaluation of currency measures to expand the long-term trade balance. The Marshall-'Lerner's (*M*-L) condition was broadened by Ref. [52], who regulated the influence of the adjustment process before the devaluation of the domestic economy.

### 2.2 J-curve theory

The J-curve is a phenomenon associated with short-term negative net export balances and subsequent long-term surpluses in response to currency depreciation [53]. [54] characterized this phenomenon as a comparative rigor of exports and imports caused by short-run foreign exchange contracts [55]. believed that later, the devaluation of the imports, exports value, and exchange rate quite signified the contract agreed by the real exchange rate before use, which was redirected in the comparative surge of import value. The determination of customs and habits and the delay in making economic decisions could be simultaneously taken as the expository pieces of evidence of the J-curve. The J-Curve points out the short run reduction of net exports, hereafter a long-term surplus aiming to cope with the depreciation of the exchange rate. As defined by Ref. [56], currency devaluation is derived from poorer. Then, it expands the balance of trade, leading to a shape related to the letter J, so there is a J-curve sensation. This phenomenon/sensation perhaps clarified that in the short run, due to foreign exchange contracts, the imports and export volume is relatively rigid [57]. [55] proved the rationality of the J-Curve phenomenon; the reason is that since the currency depreciation, the import and export value continued to illustrate the contract hinge on the preceding real rate of exchange, pondering the surge of import value comparative to domestic commodities. In addition, determining customs and habits and postponing economic decisions are also explanatory aspects of this phenomenon. [58], when evaluating and studying numerous mechanisms of the J-curve phenomenon, the findings have been mostly vague. However, after the devaluation of the domestic currency, the export prices become less expensive because the salary of domestic enterprises is relatively low according to the initial pricing. These opposite scenarios occur in the short term because the trade balance based on domestic demand for imported products will decline. In contrast, the export demand for local products will rise [18,[59], [60], [61], [62], [63], [64]]. Though scholars used different types of empirical/pragmatic methods (for a broad assessment of the works on the J-curve theory, see Refs. [65,66] for a review of literature on J-curve theory), the policies of the exchange rate with dynamic nature was not considered in previous studies.

Regarding global tourism, as tourism in worldwide destinations turns out to be more lavish, the devaluation of domestic currency beside other currencies is projected to cause the final reduction of foreign tourism [[67], [68], [69], [70]]. Nevertheless, foreign tourists from countries with devalued currencies will eventually spend more because they have already arranged their tours. Simultaneously, incoming travelers visiting countries with devalued currencies devote less, so countries' trade balance will decline promptly after the devaluation. Finally, the outward traveling demand of countries with devalued native currencies will decrease, whereas inward traveling to the countries will rise, eventually increasing the trade balance [69,[71], [72], [73]]. The influence of exchange rate on tourism demand has been extensively studied in economics tourism, for example. [68,70,74,75], tourism scholars pay less devotion to the relationship between the bilateral balance of tourism trade and the exchange rate. However, the exchange rate has an essential influence on global tourism.

Regarding global trade [67], studied the impact of the exchange rate on the tourism trade balance's overall level (USA and various other countries). Although no validation of the J-curve model was pointed out, the findings illustrated that outward tourism is further responsive to exchange rate compared the J-curve model, the findings illustrated that outward tourism is further responsive to exchange rate compared to inward tourism [72]. also used another empirical method and quarterly data to explore the linkage between the exchange rate and the US balance of trade tourism at the overall level. Their findings were related to the study of [67]. [69] analyzed the J-curve theory with empirical cointegration methods against the USA balance of tourism trade scope. Although the research results of [69] haven't endorsed the J-curve theory, the outcomes displayed that devaluation of the US dollar expands the US balance of tourism trade. The importance of these two theories has been empirically analyzed with data sets from small countries with mixed findings.

[62] used non-linear and linear ARDL co-integration techniques to explore the influence of the rate of exchange devaluation and rises on the balance of tourism trade among the UK, Canada, USA, and Mexico, based on monthly datasets during the span from January 1996 to June 2017, so there are 258 observations in each country. The results showed that the depreciation of the US$ consequently upturns the trade balance between the USA, UK, and Mexico. Even though the increase of the US dollar has worsened the two-sided balance of trade tourism between the USA, UK, and Canada, in the long term, this will not eventually influence the bilateral trade of tourism between the USA and Mexico. These findings deliver contrary indications to the J-curve theory and support the hypotheses of the Marshall-Lerner (*M*-L) condition. Hypothetical and strategic significances are raised through J-curve theory, *M*-L condition, tourism, and global trade.

[76] studied the influence of the rate of exchange fluctuations on exports, trade balance, and imports of SSA economies. The findings revealed a significant linkage between imports and exchange rate fluctuations. The outcomes recommend that exchange rate devaluation may have little or no influence on imports. Their study recommends a non-significant correlation between exchange rate fluctuations and exports. This means that devaluation of the exchange rate perhaps does not boost exports. The research moreover proposes the rate of exchange changes and the balance of trade have a negative relationship.

[77] used the pooled mean group estimator of the dynamic heterogeneity panels method to statistics from 11 SSA economies from 1993 to 2014. This research finds insignificant influences of exchange rate volatility on imports. However, for exports, the research discovered a negative impact in the short term, stable with the above observation, but in the long run, a significant impact.

[18] used time series data from 12 African economies to examine the influence of increased rate of exchange fluctuations on global imports and export flows. These data covered most African countries from the early 1970s to 2014 or 2015. By calculating the different impacts of the real rate of exchange fluctuation on exports and imports export, the authors distribute their work into diverse sections, aiming at short and long-term exploration. This research implied the ARDL bounds methods and discovered that although exchange rate fluctuation impacts the trade flows of many economies in the model, the short and long-term impacts were limited to exports of five economies and imports of one economy. The world and domestic economic activity level was acknowledged as the main factor of exports and imports.

Nevertheless [22], applied linear dynamic panel data (DPD) and System Generalized Methods of Moment (*S*-GMM) to investigate the linkage between remittance and real effective exchange rate (REER) for 32 economies from 2006 to 2016. The outcomes show that with the per capita remittance upsurge by 1 %, the REER of such economies increases by 0.103 %, which fades the attractiveness of these countries and provisions the presence of Dutch disease. The authors find that the Dutch disease occurs in economies where remittances account for a low proportion of GDP (under or up to 1 %). At the same time, for economies with high ratios, remittances lead to devaluation of the REER. In addition, considering the types of exchange regimes in various countries, the research results show that fluctuating exchange rates can inhibit the increase of real exchange rates due to capital flows. Further, Dutch disease occurs merely in economies with low exports due to the influx of remittances.

[78] assessed the significance of remittances to economic growth by applying the dynamic factor model, Panel data estimator, and the newly developed overall financial development index to investigate 61 particular emerging nations with the data set from 1970 to 2010. Since there is no universally recognized appraisal of the financial development sector, he calculated an index of financial conditions overall. Influencing economic growth was formed through an unobserved component model to define the importance of the finance sector as a mode of transmission for remittances to influence the economy's growth. He disclosed that the better a nation's finances, the more remittance will have a slighter effect on the economy's growth. Remittances can promote growth, but only when the financial development level is small will the impact be positive. These results were consistent with.

[79] prior research, where the shocks of remittances on real exchange rate were assumed to weaken 'Vietnam's competitive capacity. The findings revealed that, with the huge remittances, Vietnam is confronting a sign of the Dutch disease effect. The author points out that a 10 % increase in remittances has led to a 3 % increase in the real exchange rate effective, weakening Vietnam's competitive capacity [80]. studied the causality relationship between economic growth and imports in G7 countries, India and China. They applied the short and long-term frequency domain Granger causality test. The conclusions of this research showed that there's a high and low-frequency domain certification of the two-directional causality relationship between economic growth and imports. The imports and growth of most countries show signs of being relative in the short and long run.

Based on data from 2000 to 2021 [81], examined the relationship between economic growth and imports from China. Therefore, the qualitative research method is applied to investigate the causal relationship variables and their influence on the economy. The null hypothesis of the research study denoted that imports had a major influence on the growth rate in China. The hypothesis of this study was repulsed via the Granger causality analysis, while an uni-directional relationship was identified. However, the additional investigation was piloted using a VAR model involving important variables, such as exchange rate, inﬂation, and bank rate between the Chinese and US dollar. The impulse responses of the model were consistent with the economics theory, and findings indicated that the import had a negative relation with GDP. In contrast, the GDP had an optimistic influence on imports.

## 3 Data and methodology

### 3.1 Data source and variables

This research examines the nexus among economic growth (GDP), trade openness, exchange rate, agricultural output, and remittances in E7 (Russia, China, Turkey, Brazil, Indonesia, India, and Mexico) economies, covering the data from 1990 to 2020. All the statistics are downloaded from the World Bank, and the summary statistics (Table 2) were calculated before taking the natural logarithms. The data used for the study is shown in Table 1.

**Table 1:** Variable description.

*Variables*	Symbols	Proxy used	Source
Gross domestic product	*GDP*	GDP per capita (current US$)	WDI,2022
Exchange Rate	*EXC*	Broad money (% of GDP)	WDI,2022
Remittance	REMI	Personal remittances received (current US$)	WDI, 2022
Trade	*TRA*	Trade (% of GDP)	WDI,2022
Agriculture	AGRI	Agriculture, forestry, and fishing, value added (% of GDP)	WDI,2022

### 3.2 Descriptive analysis

As a result of the summary statistics, according to Table 2, the panel dataset's mean and median values for the variables fall between the maximum and minimum values, demonstrating a strong inclination towards the normal distribution. Positive skewness permeates every variable. The kurtosis statistics revealed that some indicators (GDP and agriculture) were platykurtic, showing that their distributions were flat compared to a normal distribution (values are less than three). However, the values of other variables are higher than three, showing that the variables are leptokurtic. The Jarque-Bera statistics demonstrate that the series is non-normally distributed because the p-value is less than 5 %.

**Table 2:** Summary statistics.

Statistics	GDP	EXC	AGRI	REMI	TRA
Mean	4945.5	1220.0	10.46	1.09	44.48
Median	3637.31	9.13	8.173	3.82	46.70
Maximum	15974.64	14582.20	27.33	8.33	110.57
Minimum	301.15	3.00	2.92	1.02	15.15
Std. Dev.	3950.57	3338.3	6.54	1.68	15.65
Skewness	0.636	2.70	0.69	2.54	0.38
Kurtosis	2.233	8.95	2.46	9.33	3.87
Jarque-Bera	19.964	586.07	20.23	597.46	12.47
Prob.	0.00	0.00	0.00	0.00	0.00
Obs.	217	217	217	217	217

### 3.3 Methodology

Before analyzing the stationarity of economic growth, trade, exchange rate, agriculture, and remittances, this research article uses the cross-sectional dependence (CSD) test of LM [82] to examine the presence of CSD in the panel database on the null hypothesis of non-cross-section dependence/correlation. The 2nd generation unit root test is appropriate to assess the stationary/stability of the determinants/variables when panel data have CSD. Since then, we have used the enhanced version of the cross-section introduced by Ref. [83] to conduct the panel unit root test. This method illustrates the CSD of the string and the homogeneity of the slopes through states. In addition, our research adopted [15,16,[84], [85], [86], [87], [88], [89], [90], [91], [92]], the panel causality test [93], and the frequency bases panel causality test of [94] to investigate the link between remittance, economic growth, agriculture, exchange rate ﬂuctuation and trade in the E7 economies.

To this end, we use our major method [93] to test the causality between variables. Panel causation analysis does not need cointegration analysis and pre-test for panel unit root [86,95] and panel frequency domain test [94]. Our panel unit test [93] can explain the heterogeneity of the determinants applied. However, we choose the D-H test based on its three benefits against present approaches: (i) it takes into consideration the dependency of cross-section, (ii) the time aspect and cross-section correlation size are irrelevant, and (iii) valid outcomes are obtained in the unstable panel.

#### 3.3.1 Cross-sectional (CDLM) dependence test

Because this research focuses on panel data, there is a high probability of correlation between the cross sections. Because of the internationalization and growing amalgamation between economies, the impact of one country may transfer to other countries. Therefore, the analogy between cross-section elements is anticipated. Overlooking this possibility might result in ambiguous and prejudiced findings [96,97]. The first stage in the study is to check whether the cross-section elements between economies are dependent. Therefore, we use the [82] LM test, which is calculated by equation (1):

(1) $$CD_{LM}=\sqrt{\frac{1}{N\left(N-1\right)}}\sum_{i=1}^{N-1}\sum_{j=i+1}^{N}\left(T{\hat{\rho }}_{ij}^{2}-1\right)$$

CD_LM_ test was introduced by Ref. [82]; it was observed that whenever the cross section (N) is greater than the time period (T), the error will happen [98]. Therefore [82], claimed that when N is significant, and T is small, a new cross-section dependency test needs to be conducted, presenting the small sample attributes [82]. conducted the following tests on cross-sectional dependency on equation (2):

(2) $$CD=\sqrt{\frac{2T}{N\left(N-1\right)}}\left(\sum_{i=1}^{N-1}\sum_{j=i+1}^{N}{\hat{\rho }}_{ij}\right)$$

where ${\hat{\rho }}_{ij}$ represents a correlation between the errors. The $H_{0}$ and $H_{1}$ hypothesis operated for the cross-section dependency test is:

$$H_{0}:\text{Cov}\left(u_{it},u_{ij}\right)=0，\;\text{There}\;\text{is}\;\text{no}\;\text{cross}-\text{section}\;\text{dependency}$$

$$H_{1}:\text{Cov}\left(u_{it},u_{ij}\right)\neq 0\text{,}\text{There}\;\text{is}a\text{cross}-\text{section}\;\text{dependency}$$

If the presumption of $H_{0}$ is accepted based on the tests carried out in the analysis, the first-generation unit root test shall be used because it does not have a cross-sectional dependence. The second generation unit root test is then used if the $H_{1}$ hypothesis is not rejected [99].

#### 3.3.2 Panel unit (CIPS) root test

The second stage is to verify the stationery of the statistics for each variable; however, the 2nd generation unit root test will contemplate the possible issue of cross-sectional dependency. The 1st generation unit root tests, like [[100], [101], [102], [103]], PP, LLC, ADF, and IPS test, respectively, disregarded the cross-section dependency. The recently developed 2nd generation unit root test (CIPS) has begun considering this issue. In this context [104], extended the augmented Dickey-Fuller (ADF) regressions with the cross-section lag lengths averages and a single series first differences in equation (3):

(3) $$\Delta Y_{i,t}=a_{i}+b_{i}Y_{i,t-1}+c_{i}{\bar{Y}}_{t-1}+di\Delta {\bar{Y}}_{t}+\varepsilon_{i,t}$$

where Δ illustrates the diverse operators, Y is the assessed variable. ${\bar{Y}}_{t}=\frac{1}{N}\sum_{i=1}^{N}Y_{i,t},\Delta {\bar{Y}}_{t}=\frac{1}{N}\sum_{i=1}^{N}\Delta Y_{i,t}$ and $\varepsilon_{i,t}$ represent the error term. The augmented CIPS test introduced by Ref. [104] is subjected to the DF regression in equation (4):

(4) $$\text{CIPS}=\frac{1}{N}\sum_{i=1}^{N}\text{CAD}F_{i}$$

where $\text{CAD}F_{i}$ is the Dickey-Fuller statistic of the cross-section in Equation (3). According to the CIPS panel unit root test, the H_0_ hypothesis represents the unit root, and the H_1_ hypothesis represents stationary [104].

#### 3.3.3 D-H causality test

The panel causality of [93] was used to test the nexus among the variables (economic growth (GDP), trade openness, exchange rate, agricultural output & remittances). The variable x is called ‘Granger-cause’ if and only if the prediction of y is greater than the older values of x and the preceding values of, instead of failing to do so [105]. This method [106] supports overcoming cross-section dependency and heterogeneity regarding alternate causal tests. Similarly, this method can be used no matt-resilient; it can be used whether N and T are greater or smaller than each other.

Moreover, this method [93] is appropriate in unstable panels. According to Monte Carlo simulations, the test statistics were comparatively strong in the limited data sample and cross-section dependence [93]. The following Equation (5) is used to check the causality between variables x and y :

(5) $$y_{i,t}=\alpha_{i}+\sum_{k=1}^{k}\gamma_{i}^{\left(k\right)}y_{i,t-k}+\sum_{k=1}^{k}\beta_{i}^{\left(k\right)}x_{i,t-k}+\varepsilon_{i,t}$$

where $\gamma_{i}^{\left(k\right)}=$ lag parameters, $\beta_{i}^{\left(k\right)}=$ slop parameters, k= lag length. $\gamma_{i}^{\left(k\right)}$ and $\beta_{i}^{\left(k\right)}$ show the variance between the unit and $\beta_{i}=\left(\beta_{i}^{\left(1\right)},\beta_{i}^{\left(2\right)},\ldots ,\beta_{i}^{\left(K\right)}\right),\alpha_{i}=$ individual fixed effects.

The hypothesis is as shown in equation (6) below:

(6) $$\begin{array}{c} H_{0}:\beta_{i}=0,\forall i=1\text{,}\ldots ,N\\ H_{1}:\beta_{i}=0,\forall i=1\text{,}\ldots ,N_{1}\\ \beta_{ii}\neq 0,\forall i=N_{1}+1\text{,}N_{1}+2\ldots ,N \end{array}$$

The hypothesis of the D-H test supposes that there's no causality in the panel contrary to the alternate hypothesis that indicates the causality exists in one cross-section unit. The Wald statistic of all panels is calculated by the mean of the individual Wald statistics of each -cross-section, as shown in Eq (7).

(7) $$W_{N,T}^{\text{Hnc}}=\frac{1}{N}\sum_{i=1}^{N}W_{i,T}$$

#### 3.3.4 Panel causality frequency domain test

Granger causality method allows determining whether the lag value of the second variable can be added to the autoregressive framework equation of the variable to expand the prediction of subsequent variables. The Granger causality method can test the influence of the lag value of subsequent variables through the basic Wald test. While the Granger causality method classifies the famous economic methods, it has certain shortcomings. For instance, we ‘can't check the presence of the Granger causality at diverse frequencies [107]. presented the Wald test to evaluate the presence of frequency domain Granger causality; applying this technique, we can study the change of time series with frequency rather than time domain, where the variance changes with time [108]. While the Granger causality test of [109], a detailed form of the [107], has been widely utilized in scientific studies, as far as we know, due to the lack of data, no research has been conducted on economic growth, exchange rate and remittance of E7 countries. In the present research, we overwhelmed this disadvantage and adopted [94] multi-countries technique in the frequency domain. The research pursued by Refs. [92,95] uses the [94] panel causality frequency domain test. We apply the subsequent seemingly unrelated equation (8):

(8) $$X_{i,t}=\sum_{j=1}^{p}\beta_{i,j}X_{i,t-j}+\sum_{j=1}^{p}\gamma_{i,j}Y_{i,t-j}+e_{i,t}i=1,2,3,\ldots ,N$$

where $X_{i,t}$ and $Y_{i,t}$ are the determinants of the economy at time. The error term of economy i at time t is $e_{i,t}$. The lag span is p, and the number of economies is N. Because $\beta_{i,j}$ and $\gamma_{i,j}$ are the definite economies, the heterogeneity taken between economies is in the framework. We applied a feasible generalized least squares (FGLS) estimator to evaluate the seemingly unrelated (SUR) framework. To investigate $H_{0}$ (yt ‘doesn't granger cause xt at the frequency w), we applied the subsequent limitations on the null hypothesis in equations 9 and 10:

(9) $$|\sum_{j=1}^{p}\gamma_{i,j}\;\cos\left(jw\right)=0|i=1,2,\ldots ,N$$

(10) $$|\sum_{j=1}^{p}\gamma_{i,j}\;\sin\left(jw\right)=0|i=1,2,\ldots ,N$$

We estimated these limitations by an incremental $R^{2}$ estimated test in equation (11):

(11) $$R_{I}^{2}=R^{2}-R_{*}^{2}$$

where $R_{*}^{2}$ and $R^{2}$ display the McElroy $R^{2}$ values of the restricted and unrestricted seemingly unrelated regression (SUR) framework, correspondingly. We reject the $H_{0}$ at frequency w in all economies at level α if equation (12):

(12) $$R_{I}^{2}>F_{\left(2N,N\left(T-2p\right),1-\alpha \right)}\frac{2N}{N\left(T-2p\right)}\left(1-R^{2}\right)$$

Here, $F_{\left(2N,N\left(T-2p\right),1-\alpha \right)}$ illustrates the α critical value (CV) of the F-statistic.

## 4 Results and discussions

Here, we put forward the outcomes of the research. First, this research assesses the CSD of the data set, subject to the [82] LM test, and afterward tests the stationary of the variables/determinants by applying the 2nd generation (CIPS) panel unit root test. Table 3 demonstrates the outcomes of cross-sectional correlation/dependence tests and CIPS.

**Table 3:** Cross-sectional dependence and CIPS panel unit root test findings.

Variables	LM Stat.	p-value
GDP	72.86	0.000***
Exchange Rate	57.12	0.000***
Remittance	62.62	0.000***
Trade	20.01	0.000***
Agriculture	64.93	0.000***
**CIPS Panel Test Results**		
**Variables**	**I(0)**	**I(1)**
**Test Statistics**		
GDP	−1.551	−3.882***
Exchange Rate	−0.441	−3.726***
Remittance	−2.156	−4.193***
Trade	−1.771	−4.951***
Agriculture	−2.988***	−5.401***

Note: *** implies the rejection of the null hypothesis at a 1 % significance level.

The empirical results show that the CSD is visible in the data. The effects of a shock/collision of any country in the group will also influence the others. Therefore, the CIPS introduced by Ref. [104] is implemented in this research, which reflects the CSD of the dataset. The CIPS test outcomes demonstrate that all determinants are stationary at I (1) in the 1st lag.

Because of the clear proof of CSD, this study is limited to variable stationarity checks using the second-generation CIPS unit root test. Table 3 shows that all metrics are aggregated in the same order. Also, the CSD test results are shown in Table 3. Cross-sections are dependent, as demonstrated by meaningful test statistics. These issues can lead to misleading results in standard unit root tests. Therefore, we used the method to determine the stationarity of variables. The outcomes suggest that the economies of the E7 countries are interconnected and highly interdependent. When an international or regional economic shock occurs in one country, other countries are also affected.

After that, we used the cross-sectional enhanced version of the panel unit root test created by Ref. [83]. This test considers the series' cross-sectional dependence and slope homogeneity across areas. Additionally, we used our main methods—the panel causality test [93], which does not need a preliminary test for panel unit root and cointegration analysis [86,95], and the panel frequency domain test [94]—to investigate the causal relationship between the indicators. A panel unit test can measure the variables' heterogeneity [93].

Table 4 displays the outcomes of D-H causality analysis, which analyzed the null of non-causality [93] of any cross-section unit beside the alternative of at least one between the cross-section units. Since the D-H analysis has no standard for selecting the optimal lag length, this research reports the Z-bar statistics of one, two, and three lag tests.

**Table 4:** D-H panel causality test findings.

Null Hypothesis	Lag = 1		Lag = 2		Lag = 3	
	W-bar	Z-bar	W-bar	Z-bar	W-bar	Z-bar
GDP → AGRI	1.769	1.123	7.212	5.569	3.565	0.214
AGRI → GDP	2.954	3.054***	2.795	0.679	4.155	0.721
GDP → EXCH	3.714	4.292	5.957	4.179	7.378	3.490***
EXCH → GDP	1.082	0.004	7.226	5.584	7.005	3.169***
GDP → REMI	1.656	0.939	5.788	3.992	7.083	3.236***
REMI → GDP	2.811	2.820***	3.703	1.684*	6.155	2.439**
GDP → TRA	3.168	3.403***	10.23	8.919***	10.19	5.909
TRA → GDP	2.691	2.624***	2.159	−0.024	4.674	1.167

Note: ***, **,* denotes rejecting the null hypothesis at 1 %, 5 %, and 10 % significance levels.

The findings illustrate that there is no causality between economic growth (GDP) and agriculture, even not in 3-lags. Nevertheless, there is a one-directional causality between agriculture and economic growth at 1st level difference. However, a bi-directional causal relationship between economic growth and exchange rate at 3rd level difference. The outcome of the link between economic growth and remittance shows two-directional causal at 3rd lag length. When we calculated the relationship between economic growth and trade, there was a two-way/bidirectional causal at 1st level difference. Hence, our research findings confirm the results of [15,48,64,76], proving a critical nexus between exchange rate and trade. This proposes that GDP can be stimulated by contemplating the maneuvering of national currency. Our research results tend to endorse the presence of the J-curve in E7 countries, as discussed in the presence of the two-way relation between exchange rate, trade, and economic growth (GDP). Our findings oppose the findings of [47,52,110], who pointed out that the J-curve theory fails to gasp between the USA and its business allies like Mexico and Turkey.

Furthermore, the trends of the causality between variables are explored at a frequency level of 2.5 (short), 1.5 (intermediate), and 0.5 (long run). In short, 0.5 denotes permanent, and 2.5 illustrates temporary causation. Comparing the results with the findings of the [93] technique and [94] panel frequency analysis, Table 5 showed two-way (permanent) causality and one-way/uni-directional (temporary) causality between economic growth and remittance. However, bidirectional (temporary & medium) and uni-directional (permanent) causal relationships exist between agriculture, trade, and economic growth. Moreover, the uni-directional (medium & temporary) causality exists from the exchange rate and GDP. This shows that E7 economies can promote their agricultural export by strictly controlling imports and export subsidies under strategic control.

**Table 5:** Croux and reusens panel frequency causality test results.

Null Hyp.	Freq.			*C*-Value
	W = 0.5 Long run	W = 1.5 Medium run	W = 2.5 Short run	10 %
GDP → REMI	0.003*	0.003*	0.004*	0.008
REMI → GDP	−1.617*	1.463	1.805	0.007
GDP → TRA	0.011	0.005*	0.004*	0.012
TRA → GDP	0.000*	0.000*	0.000*	0.007
GDP → AGRI	0.012	0.008*	0.007*	0.008
AGRI → GDP	0.005*	0.002*	0.002*	0.007
GDP → EXCH	3.569	1.122	1.655	0.005
EXCH → GDP	5.943	−1.629*	−1.371*	0.007

Note: * denotes rejecting the null hypothesis at 10 % significance levels.

## 5 Conclusion and policy implication

The relationship between economic growth (GDP), trade openness, agricultural output, remittances, and exchange rate are investigated herein through both DH analysis and in the Croux and Reusens frequency domain in E7 (China, Turkey, Brazil, Indonesia, India, Russia, and Mexico) economies covering the data from 1990 to 2020. At the beginning of the study, we applied cross-section dependence analysis to obtain appropriate unit root and causality tests. The outcomes demonstrate that cross-section dependency exists in the research. Then, we continue to employ the unit root test to check the stationary of the variables. The results indicate that the variables are stationary at 1st level differences. After successfully recognizing the variables' stationary at 1st difference, we use the [93] panel causality analysis for the time domain.

Our research findings show a two-directional causal nexus among economic growth, exchange rate, and remittance at 3rd level differences. In addition, the results reveal that economic growth, agriculture, and trade haven't a significant relation at 3rd level difference. Hypothetically, such findings highlighted the significance of the undervalued exchange rate in motivating economic growth via remittance, described by *M*-L and J-Curve theory. To adapt the time domain 'model's impairment to obtain the effect of high-frequency occurrences on the data gene, additionally, we used panel frequency causal analysis introduced by Ref. [94] to place our analysis in the frequency domain. Our research findings reveal that two-way/bidirectional (permanent) and uni-directional causality exists between economic growth and remittance.

Furthermore, our findings illustrate bidirectional/two-way (temporary) and medium causality from economic growth, trade, and agriculture. There is no causality between economic growth and exchange rate; in contrast, one-way/uni-directional (medium and temporary) causality occurs between exchange rate and economic growth. Our findings again endorse the J-Curve and *M*-L theory. This further explains the practice of the frequency-domain technique because the time-domain technique could not confirm the findings.

Our research findings provide some policy implications. First, our results point out that there's a two-directional nexus between economic growth, trade, remittance, and agriculture; that is, remittances cause growth in trade and agriculture, which may be harmful to the growth since it may stimulate domestic import demand, and consequently, import becomes less expensive. Imports might also significantly affect export, which becomes pretty inexpensive when concerning significantly impact on export, which become pretty cheap when concerning significantly affect export, which becomes inexpensive compared to other challenging countries. This means trade growth that will increase the economy's growth and increase the economy's growth. The findings of panel ganger causality indicate that the causality between variables is different in different frequencies. Policymakers in these economies may consider these scientific results to build effective and appropriate short-, intermediate, and long-run economic policies.

Therefore, the research suggests that policymakers must confirm that remittance ﬂow into the occupied zones by generating a profitable investment environment; otherwise, it will be used for consumption. For example, SMEs will promote economic growth by providing a favorable investment environment to encourage agriculture. In general, the gap in research regarding the effects of exchange rate fluctuations on remittance growth must be bridged to understand better the complicated relationship between economic growth, exchange rates, and remittances. Responding to these research gaps can provide policymakers with better policy options for maximizing the positive impact of remittances on economic growth in recipient countries. The government and policymakers can cooperate with financial institutes to inspire the implementation of digital networks and e-wallets, reducing the costs linked with remittance allocations. Moreover, policymakers and government should vigorously work to increase financial structure, develop banking facilities, and build a consistent payment structure. Such methods goal to decrease transaction charges, expand access to finance, and sponsor the proper flow of remittances.

To further promote the future research process of this topic, we suggest that scholars revise this relationship by using different variables methodology and select some other countries of the regions, distinguish between technical and nontechnical staff, and compare the findings extracted from this research.

## Funding statement

This research received no specific grant from funding agencies in the public, commercial, or not-for-profit sectors.

## Data availability statement

Data will be made available on request.

## Additional information

No additional information is available for this paper.

## CRediT authorship contribution statement

**Khalid Usman:** Conceptualization, Data curation, Formal analysis, Investigation, Methodology, Resources, Software, Validation, Writing – original draft, Writing – review & editing.

## Declaration of competing interest

The authors declare that they have no known competing financial interests or personal relationships that could have appeared to influence the work reported in this paper.
